# Narratives of Women Using a 24-Hour Ride-Hailing Transport System to Increase Access and Utilization of Maternal and Newborn Health Services in Rural Western Kenya: A Qualitative Study

**DOI:** 10.4269/ajtmh.19-0132

**Published:** 2019-09-23

**Authors:** Maricianah Onono, Gladys Ombonya Odhiambo, Ouma Congo, Lawrence Wandei Waguma, Titus Serem, Mildred Anyango Owenga, Pauline Wekesa

**Affiliations:** Kenya Medical Research Institute, Center for Microbiology Research, Kar Geno Research and Policy Hub, Kisumu, Kenya

## Abstract

Between 1990 and 2015, Kenya had a 0.9% annual reduction in maternal mortality, one of the lowest reductions globally. This slow decline was linked to the relatively low utilization of delivery services. We designed a mobile phone–enhanced 24-hour transport navigation system coupled with personalized and interactive gestation-based text messages (MAccess) to address maternal child health service utilization. The primary purpose of this analysis is to explore the ways in which pregnant and postnatal women made decisions regarding care-seeking for pregnancy and childbirth services, the processes of getting care from home to the hospital as well their perceptions on how the MAccess intervention affected their pregnancy and childbirth care-seeking and utilization experience. We conducted semistructured, individual interviews with 18 postpartum women. Participants were purposively sampled. Interviews were audiotaped, transcribed, and analyzed using thematic analysis. For participants in this study, all three delays interacted in a complex manner to affect women’s utilization of pregnancy and childbirth services. Even though women were aware of the benefits of skilled birth attendance, other health system factors such as opening hours, or health workers’ attitudes still deterred women from delivering in health facilities. The MAccess innovation was highly acceptable to women throughout pregnancy and childbirth and helped them navigate the complex and layered individual, infrastructural, and health system factors that put them at risk of adverse maternal and newborn outcomes. These findings emphasize that an integrated approach, which addresses all delays simultaneously, is important for reducing perinatal morbidity and mortality.

## INTRODUCTION

Annually, 303,000 women die from pregnancy-related complications, and almost all of them occur in developing countries, particularly in Africa and Asia.^1,2^ Approximately 60% of the maternal deaths are due to sepsis, hemorrhage, hypertensive disorders, obstructed labor, and unsafe abortion.^3,4^ In addition, for each woman who dies as the direct or indirect result of pregnancy, a significantly higher number experience a life-threatening complication that will require attention of skilled obstetric caregivers to prevent morbidity and mortality.^5^ Nearly 10% of mothers suffer a maternal complication during pregnancy or in the intra-partum period, and up to 40% may have morbidities post-birth that are attributable to the pregnancy or birth.^4^ Most of these complications are treatable and preventable during antenatal care (ANC) and if births are overseen by skilled birth attendants.^3^ Furthermore, neonatal and maternal mortality and morbidity are closely linked. About one-third of neonatal deaths occur during the first twenty-four hours of birth.^6^

Health facility delivery is a critically recognized strategy in preventing maternal and neonatal deaths.^7,8^ Unfortunately, in many developing countries, the rates of health facility delivery remain low. In sub-Saharan Africa, for example, it is estimated that up to 40% of births occur in the absence of a skilled birth attendant as compared with only 40% in the developed countries.^8^ The proportion of women delivering without a skilled birth attendant is disproportionately higher in women living in rural areas. The main factors that contribute to this disproportionate utilization of skilled birth attendants include limited geographic access to health centers, limited number of health centers with emergency obstetric care provision,^9–11^ cost of health-care services, lack of female autonomy, time available to access health care,^12^ and myths and misconceptions about health facility delivery.^13^ These factors are often summed up using the “three delays” model developed by Thaddeus and Maine^14^; deciding to seek health care (Delay 1), in accessing formal health care (Delay 2) and in receiving quality health care (Delay 3).

Kenya is one of the twenty-four countries with the highest maternal mortality rate. Between 1990 and 2015, Kenya showed one of the lowest annual maternal mortality rate reductions of 0.9%.^15,16^ This reduction was insufficient to meet the Millennium Development Goal 5^15,16^ and needs to be accelerated to achieve the Sustainable Development Goal 3, which aims to reduce the global maternal mortality ratio to less than 70 per 100,000 live births before 2030.^17^ This slow decline was linked to the relatively low utilization of delivery services. Approximately 39% of Kenyan women still deliver away from skilled help offered at health facilities, often choosing to deliver under the care of traditional birth attendants (TBAs).^18^ In response, the Kenya government abolished user fees in all public health facilities^19^ with an aim of promoting health facility delivery service utilization and reducing pregnancy-related mortality in the country, especially in the poor and vulnerable groups as has been demonstrated in the literatrure.^20^ However, despite this positive move, delays 1 and 2 contribute to two-thirds of the neonatal deaths and nearly 80% of maternal deaths. As such, there is an urgent need to develop innovative, contextually appropriate multifaceted interventions that can simultaneously address delay 1 and delay 2 to reduce maternal mortality in Kenya.

The overall aim of the MAccess project was to contribute to a reduction in maternal and newborn mortality in rural western Kenya by developing a novel mobile phone–based tool with the following suites: 1) free SMS-based service that sends personalized trimester-based texts to mothers and reminders to use ante- and postnatal care services; 2) interactive chat service known as m-convo; and 3) 24-hour transport navigator system, through which women can request for transport pick-up and the navigator service will link her to the nearest fastest, most reliable, and available driver or rider and automatically relay the information to the woman, a community health worker (CHW), and the linked health facility of the estimated pick-up time and arrival to the health facility. The primary purpose of the qualitative analysis herein was to explore the ways in which pregnant and postnatal women made decisions regarding care-seeking for pregnancy and childbirth services, the processes of getting care from home to the hospital as well as their perceptions on how the MAccess intervention affected their pregnancy and childbirth care-seeking and utilization experience.

## MATERIALS AND METHODS

We conducted a qualitative study using in-depth interviews (IDIs) as our primary data collection method. In this study, because we were interested in the personal experiences of women receiving the MAccess intervention while navigating pre- and postnatal maternal health services, in-depth interviews allowed us to obtain the lived experience of the individual women from their own perspective. The study was carried out in east Rachuonyo within Homa Bay County, which is located in rural western Kenya. Homa Bay County has one of the highest maternal and neonatal mortality rates in the country. Homa Bay’s maternal mortality rate is estimated as 583 and is very high compared with the national average of 488 and Kenya’s least-deprived counties that have rates below 200.

### Intervention description.

**Figure 1.**
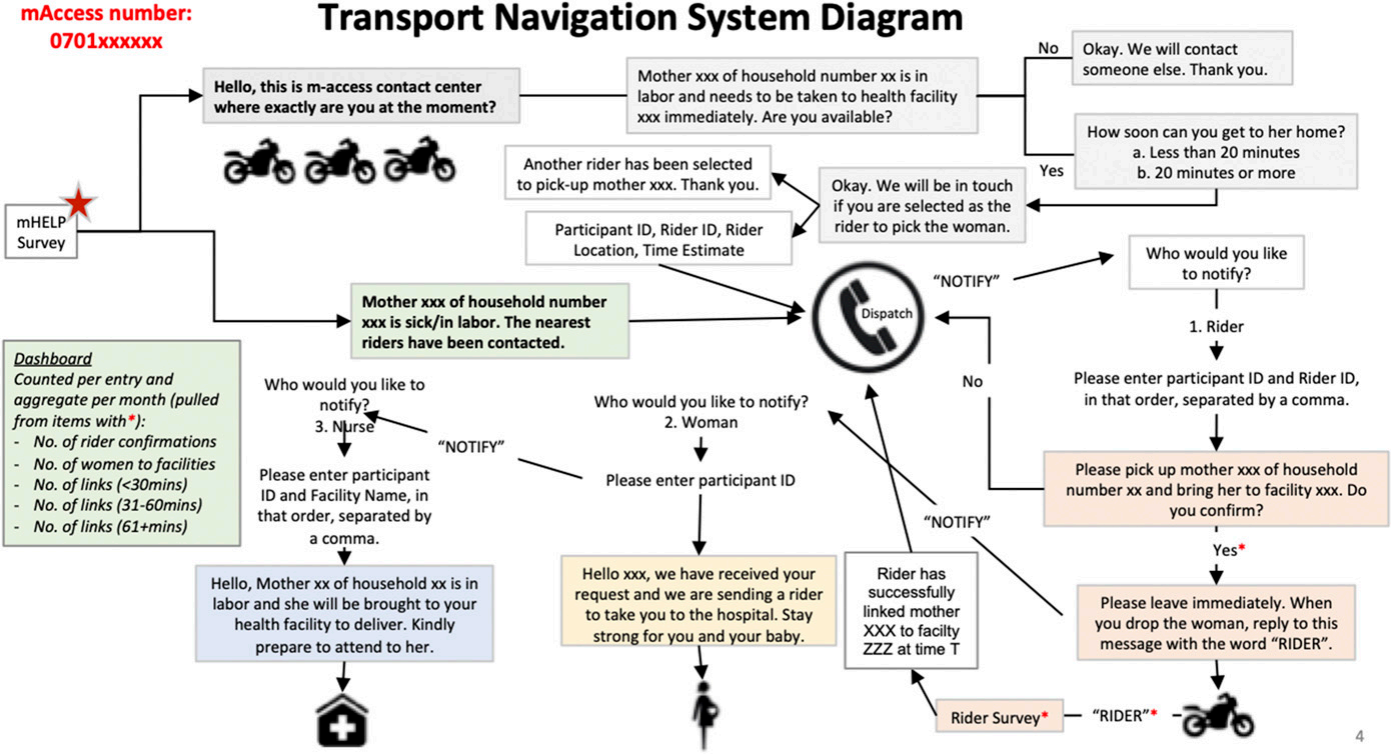
Transport navigation system starting from when the dispatch is notified. This figure appears in color at www.ajtmh.org.

The MAccess intervention comprised a two-way SMS messaging system that coordinated the sending of messages between a pregnant woman and staff. Community health workers enrolled pregnant women into the MAcess system during their routine monthly visits the homesteads as part of community maternal newborn health services. Once enrolled, each woman would automatically receive weekly messages describing what she can expect during her specific week of pregnancy. After each message, the woman was prompted to chat via text with a trained health-care worker. This was known as m-convo. The m-convo allowed the health-care worker to follow up with the woman through SMS text messaging until the woman’s questions were resolved. The woman was also able to SMS the word “mHelp” to “call for help” if she had questions, felt ill, had concerns over a danger sign, or was in labor. In the case of illness, a danger sign or labor, a central dispatcher would send a brief SMS survey to all trained motorcycle riders in the woman’s area asking a series of questions about their location and availability. Once a rider had been identified and was on his way, the dispatcher would send an SMS to the woman reassuring her that the transport is on the way and to the woman’s preferred health facility notifying them to prepare for the arrival of the pregnant women. This transport system was innovative in that, unlike the regular “Uber,” it was firmly embedded within the national and Homa Bay county government community health strategy. Within the community health strategy, CHWs are vetted for service to the communities and linked to specific community units. In the same way, motorcycle riders with a valid riders license and resident within a specific community unit applied to the community health committee in charge of community units and underwent a vetting exercise after which they are trained on their role in reducing the second delay, the importance of a quick response regardless of weather or terrain, and on community maternal newborn health. Transport fares were agreed on a community level, and each community unit had 2–3 motorcycle riders. The riders were, thus, people who the community within a specific community unit and health facility workers within the link health facility knew and who continued to participate in the monthly community dialogs. Figure 1 summarizes the transport navigation system diagram.

### Recruitment.

We used purposive sampling to recruit women in the study. The trained interviewers approached the women independently to explain the study, answer questions about the study, and guide the eligible and willing participants through the informed consent process. Women were eligible to participate in the study if they were 18 years or older and had received the standard of care and the MAccess intervention.

### Sample size.

The qualitative methodology precludes a priori sample size estimation; however, for planning of time and finances, we estimated that we would conduct IDIs with approximately 20 women who received the intervention. Emphasis was placed on ensuring that there were participants across a range of sociodemographic characteristics and parity.

### Data collection.

Trained female field workers interviewed participants in a private location at the discretion of the participant. All interviews were done in English, Dholuo, or Kiswahili. We used a semistructured IDI guide with open-ended questions to elicit reflections on what the women saw as barriers and enablers in accessing the maternal health services, perceived negative or positive impact of the intervention, and comparisons between their previous lived experiences and the current experience while receiving the intervention. The interview guide was developed using practical knowledge of the topic and existing literature. Follow-up and probe questions were, however, guided as much as possible by the participants themselves, and we allowed participants to determine the pace and content of the interview. This approach allowed for new questions to emerge during the course of the interviews, thus giving us a more thorough view and rich narratives into the women’s experiences and perspectives.

These interviews lasted approximately 90 minutes and were digitally recorded and transcribed. Informed consent was gained from all respondents before commencement of interviews. This involved an explanation of the purpose of the study, a guarantee of confidentiality relating to the information to be given, and assurance that participation would not have any negative bearing on the availability and provision of health care to them or their families.

### Analysis.

The interviews were translated into English and transcribed verbatim. Thematic analysis was chosen as our method of choice owing to its flexibility. Thematic analysis is characteristically independent of theory and epistemology and can be applied acros*s* a range of theoretical and epistemological approaches as compared with other approaches such as interpretive phenomenology.^21^ The complete data set was included in the analysis (i.e., the entire transcripts of all 18 interviews). The primary purpose of the analysis was to explore the ways in which pregnant and postnatal women made decisions regarding care-seeking for pregnancy and childbirth services, the processes of getting care from home to the hospital as well as their perceptions on how the MAccess intervention affected their pregnancy and childbirth care-seeking and utilization experience.

We used the six steps prescribed by Braun and Clarke^22^ as a guideline to carry out thematic analysis of the data. Data analysis was performed manually and continuously during the data collection period. We first familiarized ourselves with the data by reading and reading the transcripts and noting the initial ideas. Once familiar with the data, we identified preliminary codes from data that appeared meaningful and interesting. We acknowledge that the codes identified were influenced by background literature and the researchers’ experiences and values.^23^ We used comparing and contrasting techniques^24^ to identify and define codes, assign data to different codes, and search for atypical data that did not fit a particular code. This process led to the identification of broad themes from the data. Transcripts were then coded a second time and phrases that represented similar themes were further refined and clustered together into specific themes, which were then defined. Complete text from the identified themes was analyzed. Both reflective discussions and narratives of positive, difficult, and meaningful aspects of care-seeking were analyzed. Finally, relevant data were extracted according to the defined themes, and typical statements were used for citation. Two coders independently did the coding. Discrepancies were discussed with other researchers for feedback until consensus was established.

## RESULTS

### Participant characteristics.

A total of 18 participants were interviewed between November 11 and December 10, 2017. On average, the women were 27 years old, married, and multiparous with an average of two previous live births. Of the 18 participants, three had some primary level of education, seven had some secondary education, four had completed secondary school, and one had college education. All participants had hospital delivery with an average of one live birth as an outcome.

From the interviews, three main themes emerged. These were 1) presence or absence of decision-making support, 2) accessibility of health facilities and, 3) quality of care at health facilities. These themes align perfectly with the “three delays” model developed by Thaddeus and Maine^14^ to better understand the barriers to access and utilization of pregnancy and childbirth services. We also highlight through rich narratives, the mitigating role that an intervention such as MAccess can play. Quotes were selected because they were typical across most women. Names provided are pseudo-names.

### Delay 1: decision-making support.

Effective and prompt decision-making is essential for reducing delay to seek care during labor and delivery. However, decision-making for pregnancy and childbirth service care-seeking is a complex behavior influenced by individual, family, societal, access, and health system factors.^25–27^ We found that despite the availability of pregnancy and childbirth services, some women were reluctant to use the hospital-based services for several reasons such as religious beliefs and overt preference for the traditional birth attendants (TBAs).

Some people are restricted by their religious beliefs, while some have faith with the traditional birth attendants (TBA) and they say that children usually die at the hospital just like they can die at the TBA. They say that they are used to giving birth at the TBA. So they just stay back and wait for the delivery time to arrive and give birth at home. There are people who still give birth at home today not because they could not reach the hospital but because they do not want to go to the hospital. (Apondo, 20 year old with 1 living child).

During ANC visits, it is recommended that pregnant women are provided with a comprehensive birth preparedness package that allows them to plan for a normal birth and anticipate and identify the actions and arrangements needed in case of an emergency.^26,28^ When correctly implemented, birth preparedness has been shown to increase skilled care at birth and timely use of facility care for obstetric and newborn complications.^29,30^ However, in many low-resource settings, less than half of pregnant women have been found to be well informed about danger signs in pregnancy and birth preparedness practices for birth and its potential complications.^28,30,31^ The women in this study corroborate these findings through their reflections on their previous pregnancies.

…. Nobody used to teach us about birth plans. I just used to wait for delivery… I didn't know of the importance of such and economically, things were tough. I really suffered because I did not have a birth plan. I felt more relieved when I gave birth to the last baby because I prepared for delivery just as I used to be told during antenatal visits. I had even saved some money, which I used to buy food after delivery. I could afford to eat well because I had saved up to KSh 2000. (Awinja, 33 year old with 5 living children)

The first two (children), I went to the clinic only thrice for the entire pregnancy period while for the third child, I went to the clinic at least five to six times. Secondly, my financial preparation for the third child was much better than the second one. I had saved at least Ksh 4000 to help in the delivery unlike in the second pregnancy when I was quite broke. I was detained in the hospital for some days because I did not have money to pay for the hospital bill so as to get discharged. In terms of baby shawls, I had two for the last pregnancy and none during the second pregnancy. I had only one pair of clothing during the second delivery while for the third I had much more pairs. (Awuor, 30 year old with 3 living children)

Past experiences also contribute to decision-making in seeking health care. Our study found that women who had had previous successful births at home by a TBA considered pregnancy and childbirth as natural processes that does not require attention of trained health workers or the associated expenditures. Women, also, reported that the relative unfriendliness of facility-based staff deterred them from coming back to deliver at the health facilities. This is in keeping with other studies in which women preferred delivery by TBAs, as the latter were perceived to be friendly and easily available.^27,28^

Other women feel that they are experienced in childbirth and so no need for spending money at the hospitals because they can give birth by themselves. For other women, the hospitals are too far away and so accessing them is difficult. Some relative of mine once told me that she knows the entire process of child delivery and also how to cut the umbilical cord, so she didn't see a reason of spending KSh 500 at the hospital for something she can do her daughter and herself. And it was her first pregnancy, so I advised her that first pregnancies are often delicate and require expert's attention but she insisted that she knew what to do and would never spend money at the hospitals. (Awuor, 30 year old with 3 living children)

They (women who go to TBAs) receive enough support from the attendant unlike in the hospitals where you are abandoned until the last minute. That happened to me too just as I explained before. In hospital people are abandoned, they only come after you have pushed the baby. This I can confirm that is very true. However, when I gave birth to my youngest baby, they treated me better. Many women say that they are scared of going to the hospital because the nurses beat women during delivery. (Auma, 30 year old with 2 living children)

Bidirectional text messages such as those in the MAccess intervention are an innovative way to expand educational opportunities for women regarding birth preparedness. In this study, it was clear that the bidirectional text messages influenced decision-making process for the mothers by increasing their knowledge on danger signs, individual birth plans, possible complications, and immediate post-delivery neonatal care. This is demonstrated in the quotes in the following paragraph, where women reveal that the MAccess intervention helped them alleviate barriers to decision-making and birth preparedness.

I have learnt about the many ways of taking care of a child during pregnancy and after delivery. I also learnt of the things to do while pregnant so as to have a healthy pregnancy as well as safe delivery and the benefits of going to the clinic. I have also learnt about having a birth plan and saving money before delivery. I have also learnt through the text messages about the importance of vaccination. They would tell you all the things you need to do during and after pregnancy…The messages that helped me most were: the ones that encouraged me to go to the clinic, the ones about the kind of foods I was to eat and those reminding me to do some exercises. They also gave me helpful answers to the questions I asked them. I used to feel abdominal pain and they told me to lie on back and after a short while the pain went away. They were very helpful. I also asked what to do in case I do not feel the baby play for three consecutive days and they advised me to go to the hospital. (Atieno, 33 year old with 5 living children)

Another woman acknowledged the importance of having a birth partner during labor and delivery.

…They (birth partners) are very helpful even during the day. They can take care of you as the nurse attends to other people in the hospital….She can help the nurses do a few things like bringing the nurse some tools during deliver (act as a nurse aid), the person can also offer support morally and psychologically during birth. They are very helpful. They can keep you company while the nurse is going to rest. You can also send them to get you things like drinking water. She can also help you go to the toilet. (Anyango, 33 year old with 5 living children)

A recent systematic review on factors influencing implementation of interventions to promote birth preparedness and complication readiness has recommended the need for contextualized innovations that target individual knowledge and practices and also target health systems so as to avoid mismatches between local knowledge and practices and the capabilities of the health system^29^ as was identified by the respondent mentioned in the following paragraph. The respondent narrated of a woman who died because both the family and the hospital were unprepared for the pregnancy complication:

There is a woman who lost a baby in the hands of a traditional birth attendant (TBA). It was around the time that I lost my baby. I believe that if she could have gone to the hospital, she could have received helped. The baby refused to come out but the TBA kept on trying until it was late. By the time the woman was being transferred to JOOTRH in Kisumu, the baby had died. It turns out that the baby was too big for the cervix and could not pass through the birth canal. The TBA attended to her from morning to around 9 pm in the night with no success. When came here at night so that my husband could take them to the hospital. When they arrived at the local facility, it was a difficult case that had to be referred to Kisumu. It was very sad that they lost the baby but I strongly believe that if should could have gone to the hospital early enough instead of going to the TBA, the baby could have been saved. (Awiti, 33 year old with 2 living children)

### Delay 2: accessibility to health facilities.

Geographic accessibility of health facilities strongly influences the use of maternal and child health services and the attending maternal and neonatal health outcomes in many developing countries.^9–11^ Studies show that there is an inverse relationship between distance or travel time to health facilities. A recent study in Ghana showed that the probability of facility delivery ranged from 68% among women living 1 km from their closest facility to 22% among those living 25 km away.^32^ The challenge of geographic access to maternal and child health services is compounded by the fact that there are only few health facilities that can offer basic and advanced obstetric care in developing countries. Studies in sub-Saharan Africa show that facility delivery increases if the closest facility is able to provide a higher level of emergency obstetric care.^9,10^ Unfortunately, according to an obstetric data review in four East African countries, the coverage of basic emergency obstetric care services ranged 0–1.1/500,000 population compared with the United Nations’ recommended level of 4/500,000.^33^

In line with studies cited earlier, the women in this study cited long distance to the facilities of their choice, little or lack of money to pay the fare, and unavailability of reliable riders or taxis at night as challenges.

When you go to Gendia motorbike rider charge KSh 100 and when they wait for you, you pay an additional waiting charge of KSh 50. If you do not have that money, you won’t manage to go to the hospital…. (Aoko, 24 year old with 1 living child)

For the first two pregnancies, I would sometimes skip the indicated dates unlike in the third pregnancy where I was always punctual and would go on the indicated dates since transport was readily available. Previously, I would go on 20^th^ yet the appointment was on 8^th^ of the same month….Sometimes you can miss your appointment because you are tired and you do not have money for transport to go to the hospital…. (Awiti, 33 year old with 2 living children)

It is very difficult for us to get transport here at night…. When I went into labor when I had my first-born my husband was around, we were living together. It started at around 8 pm when I was cooking. I finished cooking, served and we ate. When we were about to sleep, I told my husband and after a while when the labor got intense we called for a motorbike, all the people we found refused to come because it was nighttime. He had so many of their contacts in his phone but nobody was willing to come. We even thought of walking but the hospital is far and it could have not been safe either. Everyone went to bed worried. It was a very long night. I was then taken to the hospital in the morning. (Akoth, 25 year old with 1 living child)

Although the MAccess did not improve road infrastructure, it linked women with transport providers who were reliable, given the rough terrain, odd hours of labor, and harsh weather. Women reported that the transport providers treated them with care when they were transporting them to the hospitals and they also assisted them to locate the health-care workers once they arrived in the hospitals. Motorized transportation such as motorcycles in this study has been shown to reduce referral delays for labor and delivery by up to 76%.^34^ Recent literature affirms that Mhealth solutions have the potential of bridging the second delay in maternal mortality in developing countries.^35,36^ With the increasing penetration of mobile phones in rural areas in developing countries, innovations such as the MAccess can be positioned to contribute to a significant reduction in perinatal morbidity and mortality due to the second delay by providing quick method to search and access transport. Although several studies underscore the importance of innovative rural transport networks,^37^ there are some that highlight delays in mobilizing riders who are reluctant to travel at night with a pregnant woman without nighttime allowances.^34^ These barriers can be overcome by firmly embedding emergency transport systems within community structures.^38^ The riders in this program were based on the community (not health facilities) and were selected, vetted, and transport rates agreed in the community at point of recruitment. It is likely that this fostered a sense of responsibility and accountability as demonstrated in the quote in the following paragraph.

MAccess riders respond very quickly. I was carried by different riders and they were very careful, they would ride smoothly too. The normal transport riders are very rough. I remember the one who took me to hospital that morning when I was in labor for the first time, he was very rough. He would throw me up and down over the bumps. The one who took me during the second pregnancy (MAccess rider) was very careful. I remember that it was not only dark but it had also rained. Could it have been a normal transport rider, he could have not even agreed to come because it was very muddy. He struggled on the bad road till we got to the tarmac and later the hospital. No one else could have accepted to come. MAccess motorbikes are fast, safe and reliable. (Akoth, 25 year old with 1 living child)

My first experience was quite good. I was seriously ill and unable to walk, so when I requested for transportation, the rider came very fast and helped me get to the hospital. When we arrived at the hospital, we did not find the health facility in charge. He even called the clinical officer and handed me to him when he came. Immediately I was done, I called him back and he never hesitated…. That was a great experience, the rider helped me a lot…He (my husband) asked me why I was choosy on the type of riders who would take me to the hospital and I explained to him that I only use the trained motorbike rides who belong to MAccess and have knowledge on how to handle expectant mothers on the way and even if someone delivered on the way, they would know what to do. That is why I have to use MAccess riders. (Awuor, 30 year old with 3 living children)

### Delay 3: quality of care at health facilities.

Quality of care is a major multifactorial concept in health that includes actions, interactions, and relationships between many components in the health system.^39,40^ The WHO’s quality of care framework for maternal and newborn health outlines the critical components of quality of care as processes, structures, and outcomes.^39^ Structures include physical and policy settings and the need for adequate resources. Under processes, the framework highlights that the provision of care must be evidence based, of the appropriate amount of care, that is, neither “too little, too late” nor “too much, too soon,” respectful and above all, sensitive to the woman’s experience of care.^39,40^

Women in this study perceived the facilities as the safest place of delivery that would ensure positive birth outcomes.^41,42^

…Women should give birth at the hospital. The hospital cannot let you suffer. For example, when you are bleeding at the TBA she will not help you because she doesn’t have medicine that can stop the bleeding. Also, when you need an emergency cesarean section, the TBA will not save your lives because she doesn’t have a theatre. Even in my case, I went to the hospital late after laboring for some time and when the baby was delivered she was so distressed, she had to be put in the nursery where they put her on oxygen to resuscitate her. Suppose I delivered at the TBA my baby could have died or we could have all died. The TBA cannot save the life of the baby if the mother is HIV positive. She can mix their blood when she is cutting the umbilical cord thus infecting the baby. I therefore, wish that all people go to give birth at the hospital. (Apidi, 24 year old with 1 living child)

Unfortunately, similar to other studies in low-resource settings, facilities lacked infrastructure, adequate number of staffing which led to women queuing for long hours, and being neglected to the extent that they often delivered themselves or just flat out did not attend ANC.^43–48^

I did not wish to make many trips to the hospital because walking up to Gendia, which was the hospital I preferred was challenging. I do not like the nearby health center because it is normally crowded and women spend a lot of time queuing. Lack of transport was one of the challenges that I experienced because if you go on a motorbike you have to pay for it. At the same time the nearby health center had very long queues. When MAccess riders used to pick me up and take me for antenatal care clinic at Gendia, I used to go and come back before someone who went to the Junction hospital. (Atieno, 33 year old with 5 living children)

When I arrived at the hospital during my first baby, I was put on bed and left on my own the whole day. They only came to attend to me when the worst got to the worst…. I felt neglected; they also said very nasty and mean things to me. I had labored for long but they still accused me of taking traditional medicine which make baby to be born quickly. It was my first pregnancy and I did not know anything, the more they ignored me the worst I felt. In fact, they were surprised to learn that I had given birth by myself and only came to my aide when the baby had already landed on the floor. (Adongo, 25 year old with 1 living child)

Women also reported that the providers were verbally abusive and lacked the compassion and patience to respond to their concerns during pregnancy such as absence of fetal movements. Previous studies in sub-Saharan African countries such as Ghana, Nigeria, Tanzania, and Kenya have reported a high prevalence of mistreatment and disrespect of women characterized by verbal abuse (shouting, insults, and derogatory remarks), physical abuse (pinching, slapping), abandonment, and lack of support.^49–51^ Based on these experiences, women made deleterious choices to not attend ANC, deliver at home, or delay to go to the hospitals for delivery and ANC. Previous studies have demonstrated a clear relationship between mistreatment and disrespectful maternity care and future childbirth in health facilities.^52,53^

She (nurse) would arrogantly tell me to weigh myself and take the readings to her, in measuring blood levels she told me to give out money for the tests to be conducted or else I just go back home, after all my pregnancy is none of her business and she cares less. That was so disheartening. (Atieno, 33 year old with 5 living children)

The other thing that makes women not to go the hospital is because the nurses do not show concern. They do not attend to other needs of the women even when they say that they are not feeling well. They just do routine check-ups and tell them to go home. They do not tell you what to do with your problem neither do they refer you like MAccess used to. When you text your problem to MAccess, they refer you to hospital or tell you what to do. These nurses at the hospital just do not care. They just assume your problem and tell you to come back during your next clinic appointment. Moreover, they do not care whether you come back or not...Lack of appetite, child not playing in the womb, too much weight on one side. These are problems that the nurses can solve but they do not care…When they go to the hospital they tell them about these problems but they are not given any solutions. (Apidi, 24 year old with 1 living child)

Poor communication between providers and patients is not only a deterrent for use of services but also fosters myths and misconceptions. For example, women in this study alluded to the fact that iron and folate supplements (IFAS) given during ANC could contribute to child deformity. This likely indicates that the health-care workers may not have had time to explain the importance of the drugs, and for one reason or another, the women do not feel comfortable to ask questions when they go for their ANC visits.

Some say that the kinds of drugs (IFAS & vaccines) they are given at the hospitals do have negative impacts on their children. I have a co-wife (married to brothers), who used to throw away the drugs she was given at the hospital during pregnancy. She would say that IFAS have negative impact on children. Some people say that the iron tablets do cause abnormalities in children, though we have always been taught that such tablets actually prevent the abnormalities. Whenever someone gives birth to a baby with defects from the hospital, the women say that there is no difference in home and hospital delivery. This is because they say that the women went to all her clinic appointments but still gave birth to a baby with birth defects and so there is no point in going for antenatal care. (Awinja, 33 year old with 5 living children)

Recent literature review points out that Mhealth solutions have the potential of addressing the three delays in maternal mortality in developing countries.^54^ The bidirectional SMS system offered by the MAccess mitigated the challenge of inadequate information, education, and some of the communication gap. The application was able to defray myths and misconceptions regarding facility births and use of supplements. The riders had contacts of the health providers and were able to alert them about women coming in which allowed providers to prepare to receive the mothers properly or be directed to less busy but equipped health facilities. This nontransport role of riders created a positive perception among our participants toward health-care services offered in the facilities, and hence improved the ANC attendance. On the other hand, MAccess did not address the poor attitudes of health workers toward women, which is an important deterrent to care-seeking. Regular coaching of staff, supervision and clinical audits of maternal and neonatal mortality and morbidity, integration of maternal and neonatal indicators in routine reporting, and sharing the best practices among colleagues have been recommended as ways to improve quality of maternal care at facility level.^55^ Future reiterations of the SMS system can, however, be improved to foster education and empowerment of women and their birth partners seeking services to be able to navigate mistreatment by health-care workers by using techniques such as deflecting mistreatment, improving communication, and advocating for human rights and dignity of pregnant and postpartum women.

The study limitations deserve mention. One limitation of the study is that we used purposive sampling, and the study nurses identified the potential participants. We acknowledge that in giving the nurses this “gatekeeping role” that this might have shaped the type of participants enrolled into the study, for example, by nurses selecting potential participants who were better known to them. In mitigation, we limited the role of the nurses in identifying only those participants who met the eligibility criteria regardless of their relationship and engagement with the nurses or the clinics. In addition, interviews were conducted mainly in Dholuo; therefore, cultural variations beyond Luo-speaking regions of Kenya are not represented. Further research to confirm findings and to empirically test recommended community and programmatic implications are needed.

In conclusion, in this article, we have presented narratives of pregnant and postpartum women navigating maternal and child health services before and after the introduction of a mobile phone–enhanced 24-hour Uber-like transport navigation system coupled with personalized and interactive gestation-based text messages. The goal was to unpack and describe the broad range of micro-, meso-, and macro-contextual factors that influence their care-seeking experiences and how innovative use of Mhealth can facilitate women’s navigation of the complex health system. Their narratives suggest that all three delays interacted in a complex (nonlinear) manner to affect women’s utilization of pregnancy and childbirth services. Although women were aware of the benefits of skilled birth attendance, other health system factors such as opening hours or health workers’ attitudes still deterred women from delivering in health facilities. The Mhealth innovation was found to be attractive and highly acceptable to women throughout pregnancy and childbirth, and helped them navigate the complex and layered individual, infrastructural, and health system factors that put them at risk of adverse maternal and newborn outcomes.
